# Quantification and identification of lightning damage in tropical forests

**DOI:** 10.1002/ece3.3095

**Published:** 2017-06-02

**Authors:** Stephen P. Yanoviak, Evan M. Gora, Jeffrey M. Burchfield, Phillip M. Bitzer, Matteo Detto

**Affiliations:** ^1^ Department of Biology University of Louisville Louisville KY USA; ^2^ Smithsonian Tropical Research Institute Balboa Panama; ^3^ Department of Atmospheric Science University of Alabama in Huntsville Huntsville AL USA; ^4^ Department of Ecology and Evolutionary Biology Princeton University Princeton NJ USA

**Keywords:** camera, disturbance, forest canopy, landscape, mortality, Panama, tree

## Abstract

Accurate estimates of tree mortality are essential for the development of mechanistic forest dynamics models, and for estimating carbon storage and cycling. However, identifying agents of tree mortality is difficult and imprecise. Although lightning kills thousands of trees each year and is an important agent of mortality in some forests, the frequency and distribution of lightning‐caused tree death remain unknown for most forests. Moreover, because all evidence regarding the effects of lightning on trees is necessarily anecdotal and post hoc, rigorous tests of hypotheses regarding the ecological effects of lightning are impossible. We developed a combined electronic sensor/camera‐based system for the location and characterization of lightning strikes to the forest canopy in near real time and tested the system in the forest of Barro Colorado Island, Panama. Cameras mounted on towers provided continuous video recordings of the forest canopy that were analyzed to determine the locations of lightning strikes. We used a preliminary version of this system to record and locate 18 lightning strikes to the forest over a 3‐year period. Data from field surveys of known lightning strike locations (obtained from the camera system) enabled us to develop a protocol for reliable, ground‐based identification of suspected lightning damage to tropical trees. In all cases, lightning damage was relatively inconspicuous; it would have been overlooked by ground‐based observers having no knowledge of the event. We identified three types of evidence that can be used to consistently identify lightning strike damage in tropical forests: (1) localized and directionally biased branch mortality associated with flashover among tree and sapling crowns, (2) mortality of lianas or saplings near lianas, and (3) scorched or wilting epiphytic and hemiepiphytic plants. The longitudinal trunk scars that are typical of lightning‐damaged temperate trees were never observed in this study. Given the prevalence of communications towers worldwide, the lightning detection system described here could be implemented in diverse forest types. Data from multiple systems would provide an outstanding opportunity for comparative research on the ecological effects of lightning. Such comparative data are increasingly important given expected increases in lightning frequency with climatic change.

## 1 INTRODUCTION

Humans have a long history of fear and fascination with lightning (Andrews, Cooper, Darveniza, & Mackerras, 1992; Botley, 1951; Bouquegneau & Rakov, 2010; Franklin, 1769), and scientists have been exploring the physics and biological effects of lightning for more than a century (e.g., Anonymous, 1898; Stone, 1903; also see Rakov & Uman, 2003). However, the specific role of lightning as an ecological disturbance remains one of its least‐studied aspects. This is a particularly important knowledge gap in the tropics, where lightning frequency is relatively high, but the ecological effects of lightning strikes have never been quantified at large spatial scales in real time.

This gap in our understanding of the ecological importance of lightning mainly is attributed to two logistical obstacles. First, the spatial unpredictability and temporal unpredictability of lightning constrain documentation of the immediate ecological effects of lightning strikes to serendipitous, unreplicated observations (e.g., Furtado, 1935; Orville, 1968; Tutin, White, & Mackanga‐Missandzou, 1996). Second, whereas ground‐based and satellite‐based lightning flash detection systems generate accurate flash frequency data at regional scales (Albrecht, Goodman, Buechler, Blakeslee, & Christian, 2016; Boccippio, Cummins, Christian, & Goodman, 2001), they do not provide sufficient spatial accuracy to locate tree damage caused by individual flashes (Mäkelä, Mäkelä, Haapalainen, & Porjo, 2016). Here, we describe field‐based methods that collectively enable the accurate quantification of lightning strike frequency, lightning characteristics, and lightning‐caused tree mortality at the km^2^ scale in near real time (i.e., within hours of a storm event). We focus on tropical forests, but the methods could be applied to any terrestrial ecosystem.

Discussions of lightning‐caused ecological disturbance invariably focus on trees. Indeed, lightning strikes thousands of trees worldwide each year (Taylor, 1974) and is an important agent of tree mortality in some forests (Yanoviak et al., 2015). Ecologists commonly assess lightning damage to trees via post hoc surveys (e.g., of lightning scars on trunks; Taylor, 1964, 1965). However, this approach is inherently biased because lightning effects on trees are extremely variable—from catastrophic trunk shattering (Fernando, Mäkelä, & Cooray, 2010; Stone, 1916; Taylor, 1974) to no obvious damage (e.g., Orville, 1968). Moreover, the lethal effects of lightning often are protracted (Furtado, 1935) and death commonly occurs via indirect mechanisms (e.g., beetle and fungal infestations; DuCharme, 1972; Coulson et al., 1983). Thus, a considerable fraction of lightning‐caused tree damage and mortality likely either goes unnoticed or ultimately is not attributed to lightning. Finally, field‐based forest surveys often are conducted at intervals >1 year (commonly 5 years), which greatly limits the accuracy of lightning damage assessments, especially given that lightning scars can become unrecognizable over time due to localized healing, decomposition, or secondary infections (SPY and EMG, pers. obs.).

Historically, post hoc lightning damage surveys have been most effective in temperate pine forests, in part because lightning damage to coniferous trees commonly appears as a conspicuous longitudinal stripe on the trunk (Gora & Yanoviak, 2015; Outcalt, 2008; Wadsworth, 1943). By contrast, the best available landscape‐scale data for tropical forests are limited to surveys of conspicuous lightning gaps (Anderson, 1964; Brünig, 1964; Magnusson, Lima, & de Lima, 1996). These large group mortality events presumably result from the most intense lightning flashes (although this has never been verified) and represent a small fraction of the total number of strikes in a forest. Collectively, these limitations suggest that post hoc surveys significantly underestimate lightning‐caused disturbance in forests, especially in the tropics.

Ecological assessments of lightning disturbance also are limited by a lack of information about the strike itself. Individual lightning flashes can have positive or negative polarity and differ substantially in intensity (measured as peak current) and duration (Rakov & Uman, 2003). The return strokes (the familiar, visible portion of a lightning discharge) of cloud to ground (CG) and ground to cloud (GC) flashes cause injuries to trees. Many flashes have multiple return strokes, each lasting just tens of microseconds, but some “continuing current” (CC) strokes persist for hundreds of milliseconds and likely initiate forest fires (Bitzer, 2017; Fuquay, Taylor, Hawe, & Schmid, 1972). It is possible to measure the polarity, intensity, duration, and multistroke nature of a lightning flash (Bitzer et al., 2013). However, to our knowledge, only one study has attempted to associate such characteristics with the direct effects of lightning on trees (Mäkelä, Karvinen, Porjo, Mäkelä, & Tuomi, 2009); no similar studies exist for tropical forests.

The only solution to the problems summarized above is to locate lightning strikes and to measure their characteristics as they happen. As noted above, satellite‐based optical detection and land‐based networks of electronic sensors can provide regional flash distribution data in near real time (Cummins & Murphy, 2009), but their limited spatial accuracy makes locating lightning damage difficult or impossible (Mäkelä et al., 2016). The methods described here overcome this problem and establish a basis for accurate quantification of lightning strikes at the landscape scale. This information, in combination with field‐based assessment of tree condition, and knowledge of tree traits (e.g., Gora & Yanoviak, 2015) and lightning discharge characteristics, can provide comprehensive assessment of the ecological effects of lightning in forests.

Accurately quantifying lightning‐caused disturbance is important because the frequency of CG lightning is expected to increase with climatic change (e.g., Romps, Seeley, Vollaro, & Molinari, 2014; Williams, 2005). Specifically, models predict that for each 1°C increase in average surface temperature (or each doubling of atmospheric CO_2_ concentration), CG lightning as a fraction of total lightning will increase by at least 10% (Price & Rind, 1994a, 1994b; Williams, 1992, 2005). In the wet tropics, this will likely occur via increased storm intensity, prolonged interstorm intervals, increased drying between rain events (Price, 2009), and increased lightning‐initiated fires (which currently are very rare in lowland rainforests). Models further suggest that smoke from agriculture and lightning fires will increase storm intensity and thus lightning frequency, often at locations very distant from the source fire (Cochrane, 2003; Goldammer & Price, 1998; Price, 2009; Price & Rind, 1994b). There is growing evidence that such changes are already happening (Norris et al., 2016). Thus, accurately measuring lightning‐caused tree mortality will be relevant to predicting future forest dynamics and structure under a changing climate.

The principal objective of this study was to establish a lightning monitoring network that would enable us to determine the locations of lightning strikes on a forest‐wide scale (ca. 20 km^2^), in near real time, and with high spatial accuracy (ca. 10 m). Two secondary objectives were to (1) demonstrate how ecologically relevant lightning flash characteristics (intensity, polarity, duration, and number of return strokes) can be quantified and (2) generate a standardized list of indicators that could be used to reliably identify lightning damage in the field post hoc.

## 2 METHODS

Field work for this project was conducted in central Panama on Barro Colorado Island (hereafter, BCI; 9.152°N, 79.846°W) and Gigante Peninsula (hereafter, Gigante; 9.128°N, 79.856°W). BCI is a 15‐km^2^ island administered by the Smithsonian Tropical Research Institute (STRI) and is one of the best‐studied patches of tropical forest on earth (Croat, 1978; Leigh, Rand, & Windsor, 1996). The forests on BCI and Gigante are categorized as seasonally moist, with a well‐defined wet season spanning May to December. Much of the rainfall in the area comes from storms associated with tropical low‐pressure waves. Many of the storms produce frequent lightning; BCI receives ca. 40 flashes km^−2^ year^−1^ and peak flash rates occur between mid‐July and mid‐August (from 1995 to 2012 satellite data; Christian et al., 2003). Roughly 25% of those flashes are CG lightning (Boccippio et al., 2001; Price & Rind, 1993) and thus are potentially damaging to trees. Consequently, BCI currently receives ca. 150 CG strikes per year. Some tropical lightning hotspots (e.g., the Congo, the Colombian Chocó, and Lake Maracaibo; Albrecht et al., 2016) have lightning flash frequencies ca. twice that of BCI. We focused on Panama (rather than a lightning hotspot) because it uniquely offers high lightning frequency in combination with political stability, excellent infrastructure, established long‐term forest research plots (Hubbell & Foster, 1983), and easy access.

### 2.1 Video camera network

**Figure 1:**
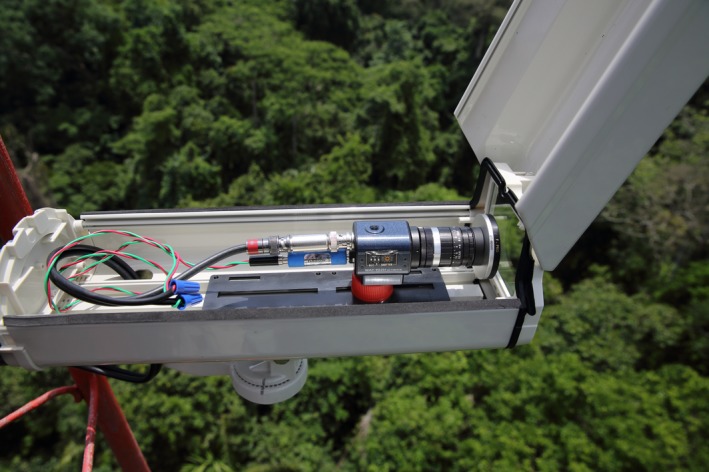
A surveillance camera mounted on one of the towers used in the video network for lightning monitoring. A 3.0 neutral density filter is attached to the front of the camera, and both the power supply and video feed cables are fitted with in‐line surge protection. The complete setup is protected within a metal box, which is open for the photograph

**Figure 2:**
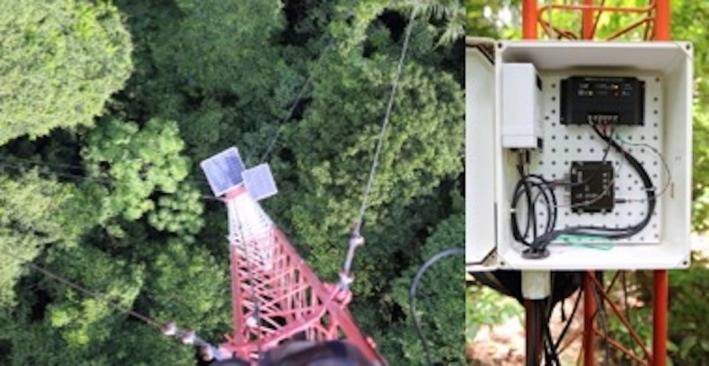
The solar panels (left) and electronic equipment (right) used to support individual cameras in the video network. The panels collectively provided up to 90 W of power to recharge a deep‐cycle 12‐V battery (not shown) during the day, while also powering the camera, GPS antenna, text overlay unit, and DVR. These latter components were then powered by the battery through the night

Measuring CG lightning frequency, distribution, and damage to individual trees requires continuous monitoring of large areas of forest canopy at resolution of <20 m, which is impossible with traditional lightning quantification methods (Mäkelä et al., 2016; Stall, Cummins, Krider, & Cramer, 2009). An alternative approach—placing a large number of electronic sensors in a forest—would provide adequate spatial resolution, but also would be logistically difficult and very costly. We established a video‐based lightning monitoring system on BCI in 2014 to overcome these problems. The system consisted of video surveillance cameras mounted on a series of preexisting guyed towers overlooking the forest canopy (Figures 1 and 2, Table 1; Kays et al., 2011). Tower height was ca. 43 m and extended >10 m above the surrounding forest canopy in all cases.

**Table 1:** Sources of equipment used in the project

Description	Manufacturer	Model number
Guyed tower	ROHN Products, LLC, Peoria, Illinois	Rohn 25
Video camera	Watec, Inc., Japan	WAT‐902H2 Ultimate
Camera lens	Fujifilm Optical Devices, Inc., Wayne, New Jersey	DF6Ha‐1B
Camera filter	Schneider Kreuznach Optics, Germany	B+W 65‐1066177
Camera box	Pelco Inc., Clovis, California	EH3512
Solar panel	SolarLand Inc., Grayslake, Illinois	SLP030‐12U
Charge controller	SolarLand Inc., Grayslake, Illinois	SLC‐NR2420A
Text overlay unit	BlackBox Camera Co., London, United Kingdom	GPSBOXSPRITE2
GPS antenna	BlackBox Camera Co., London, United Kingdom	ANT‐555
DVR	Pinecom Surveillance Inc., Covina, California	PL0067
Distance meter	Leica Geosystems, Norcross, Georgia	Disto D5

**Figure 3:**
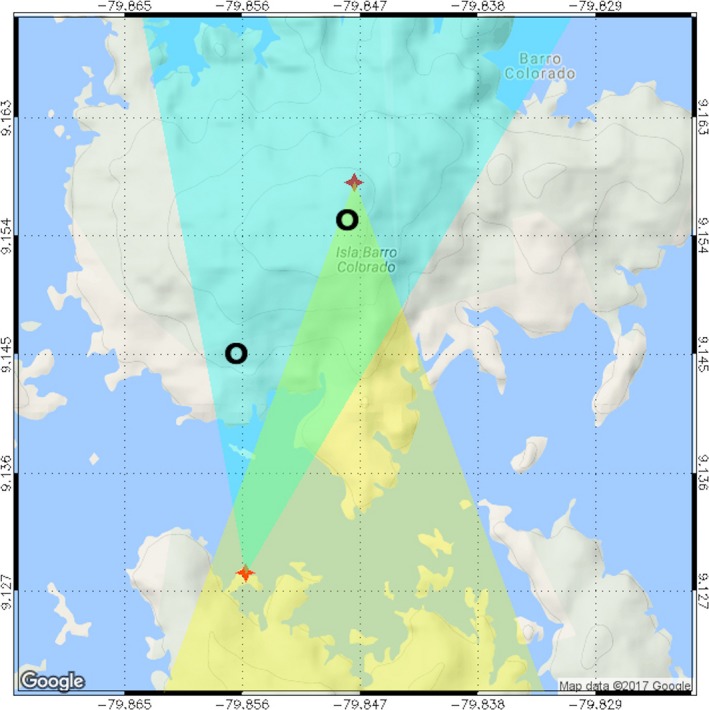
The field of view of each of two cameras used in the first iteration of the camera network (2014). Circles = approximate locations of the two strikes shown in Figure 5

**Figure 4:**
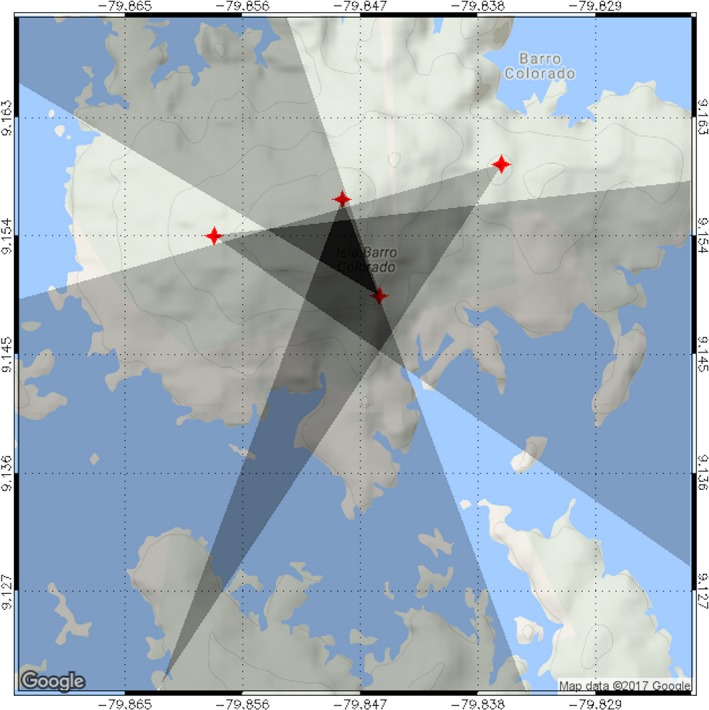
The field of view of each of four cameras used in the second iteration of the camera network (2015–2016). Under this arrangement, ca. 15% (225 ha) of BCI is viewed by at least two cameras

Each video camera was fitted with a 6 mm f/1.2 lens and a 77 mm 3.0 neutral density filter and housed within a metal protective box (Figure 1 and Table 1). The cameras operated at an interlaced shutter speed of 1/30 s with apertures ranging from f/4 to f/8, depending on local light conditions and camera orientation. Once installed, each camera recorded digital videos continuously to a DVR fitted with a 32 Gb SD memory card located in a weatherproof box at the base of each tower (Figure 2 and Table 1). The camera and recording components on each tower were powered by a 12‐V deep‐cycle marine battery charged by two or more solar panels wired in a parallel circuit to generate up to 90 W under full sun exposure (Figure 2). The preliminary system established in 2014 provided visual coverage of ca. 50% of the island by one camera and 15% of the island by two cameras (Figure 3). The system was expanded to four cameras in 2015 and 2016 to provide approximately the same amount of spatial coverage (Figure 4), while improving directional diversity and removing the logistical constraint of frequent travel by boat to Gigante. Each camera recorded video for up to 8 days before the SD card approached capacity. The amount of data recorded per minute varied based on individual camera settings, so the SD cards were removed from the DVRs and replaced with blank cards every 3 or 4 days to avoid data loss.

We used frame‐by‐frame analyses of the videos from each camera to identify lightning strikes. These analyses were conducted automatically via an IDL program that compared the brightness of each frame to the previous frame on a pixel‐by‐pixel basis (Supporting Information). Specifically, if more than one hundred pixels were brighter by a particular threshold (typically, ten bytes), then the frame was marked as potentially containing a lightning strike. Frames with brightness exceeding the threshold were saved and subsequently examined by the authors to verify the presence of lightning flashes. The SD cards collectively contained hundreds of Gb of digital video even after just 3 days, so we expedited the video processing by targeting time intervals surrounding known storm events. We identified these time intervals based on our own observations of electrical storms on BCI, and by polling other researchers residing at the station. We also used Panama Canal Authority (ACP) weather radar images to verify the timing of storm events. The radar images were captured automatically from the ACP website (http://www.pancanal.com/eng/radar/main.html) via a short Unix shell script. The program recorded the radar image every 5 min, and the subsequent image stack was aggregated, using IDL, into an MPEG‐4 video loop spanning the previous 24 hr at 06:00 every day (Video S1, Supporting Information). Upon identifying the time frame associated with a specific storm event, we extracted the corresponding videos from the SD cards for frame‐by‐frame processing as described above.

**Figure 5:**
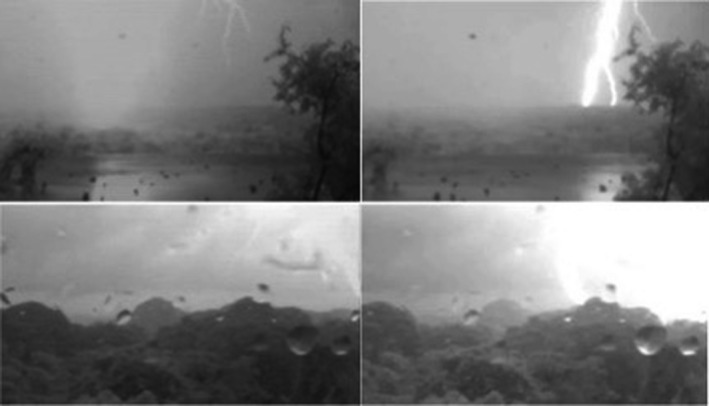
Still video frames of two lightning strikes to BCI trees on 14 August 2014, 16:14 local time. The top images were captured by the camera on Gigante, and the bottom images are from the camera on BCI. The two images on the left are of the first flash (*Example 1* in the text), and those on the right are of the second flash (*Example 2*). The field of view of each camera and the approximate strike locations for these two flashes are shown in Figure 3. All images were cropped for clarity

**Figure 6:**
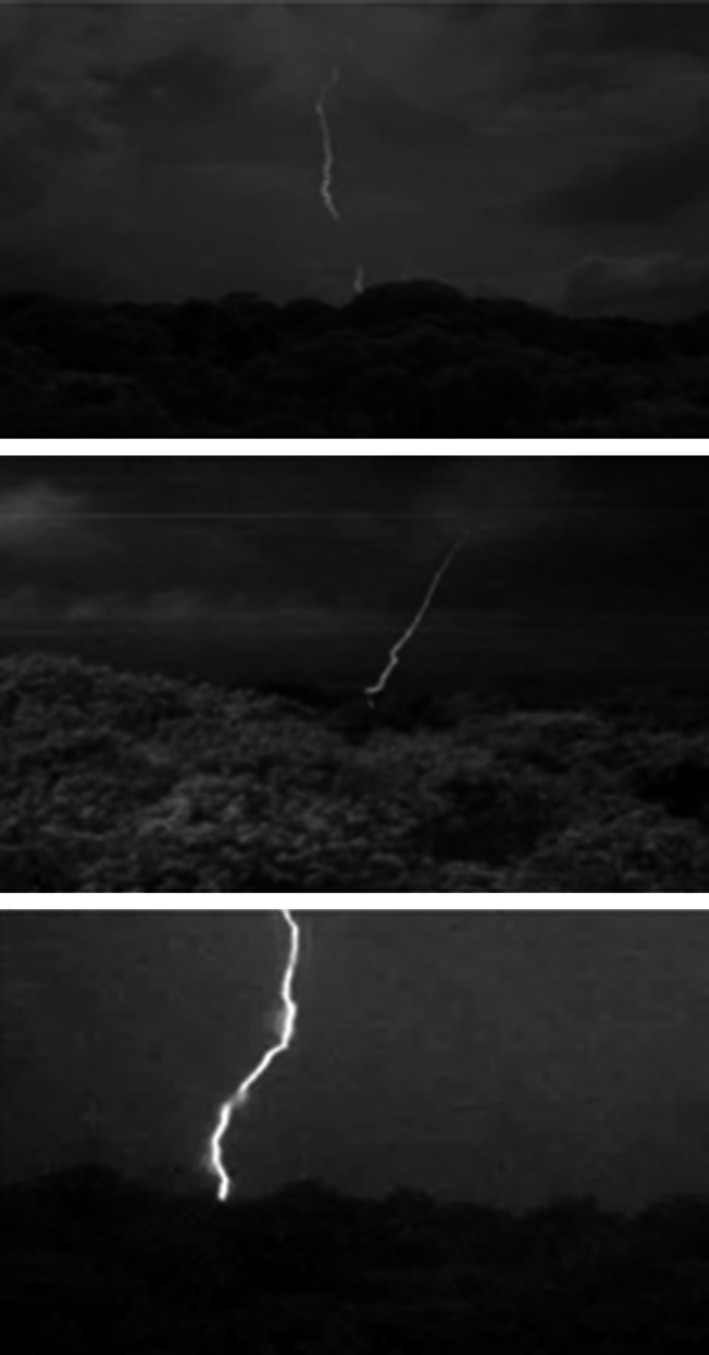
Still video frames of a lightning flash captured by three cameras on BCI on 21 September 2015, 16:18 local time. The cameras were located on towers associated with BCI trail names as follows: Zetek (top image), AVA (center image), and Drayton (bottom image). All images were cropped for clarity. Also see Figure 7

**Figure 7:**
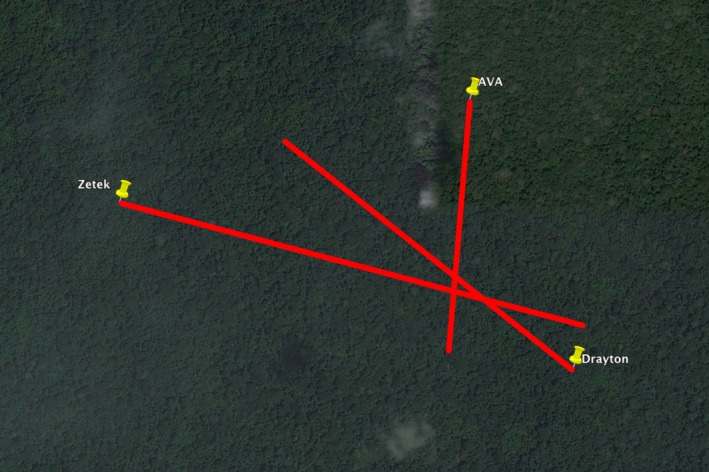
An annotated Google Earth image of BCI illustrating how the approximate location of the strike recorded in Figure 6 was determined based on the azimuth and field of view of each camera. The coordinates near the intersection of the three lines were used to locate and ground‐truth the site. The length of the AVA line is ca. 650 m

We determined the approximate locations of lightning strikes by comparing images of the same flash recorded by two or more cameras mounted on different towers (Figures 5 and 6). The azimuth and field of view of each camera provided a reliable estimate of the strike location, which was then mapped on Google Earth (Figure 7) and verified by hiking to the site. This approach enabled us to locate strike sites within a few days of the event. The successful implementation of the camera‐based system provided the logistical foundation for the development of the more sophisticated and efficient ELS system described below.

### 2.2 Electronic network

**Figure 8:**
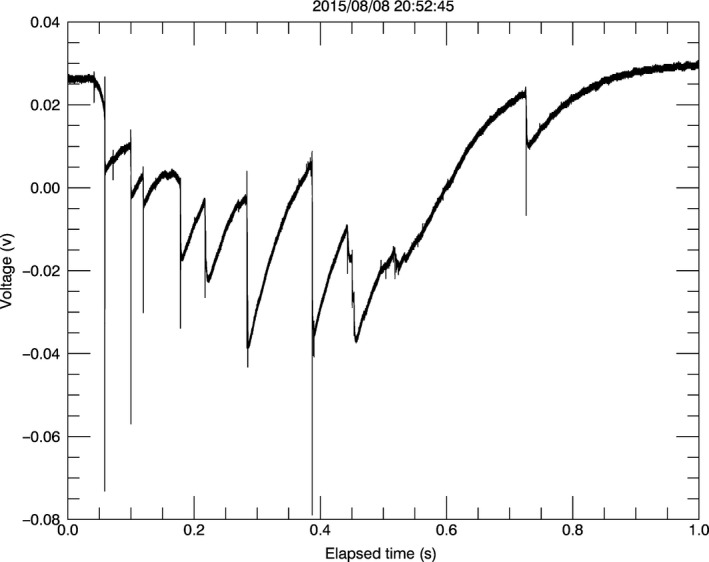
Time series of a nine‐stroke negative lightning flash recorded by the field change meter on BCI. Rapid changes in voltages indicate CG discharges and their polarity, and the amplitude of the change indicates the peak current (i.e., electrical intensity) of each discharge

In addition to the video monitoring system described above, we will deploy two advanced electronic lightning sensors (ELSs) in 2017. The sensors were constructed for this project in the Atmospheric Science laboratory at the University of Alabama in Huntsville and were tested for basic functionality on BCI in 2015 and 2016 (Figure 8). The key component of the ELS is a field change meter that records variation in electrical fields at extremely fine temporal scales (<1 μs; Bitzer et al., 2013), providing intensity, duration, and polarity for each CG flash (Krider, 1992; Figure 8). The radiation from a return stroke produces a unique signature detected by the ELS, allowing the differentiation of CG strokes from other lightning discharges. As explained below, the ELS array will provide very accurate CG flash locations for most of the study site.

Each ELS records the precise moment that a return stroke forms (generally <10 m above the strike point). The time difference between two ELS recordings is then plotted in combination with information from a single camera image of the flash. Specifically, the difference in arrival times of the radiation at each ELS yields hyperbolic curves describing possible return stroke locations. The actual location within that distribution is determined from the location of the lightning flash in the camera image and the azimuth of the camera lens axis. Collectively, this information provides very accurate tree strike locations within minutes of the event and dramatically reduces dependence on cameras.

**Figure 9:**
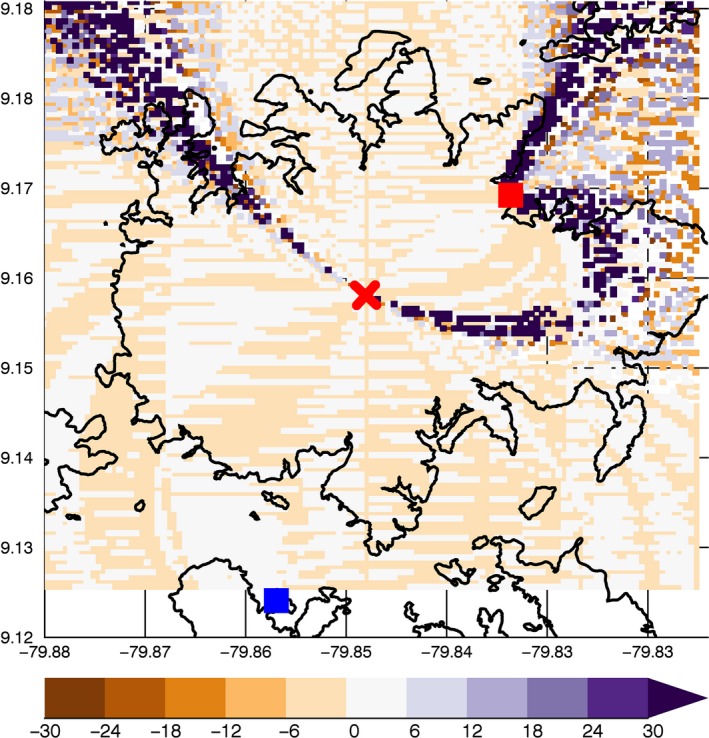
Expected spatial error (m) for lightning strike locations using the ELS network. Data from field change meters on Gigante (blue square) and the BCI laboratory clearing (red square) plus camera images from a central tower on BCI (red X) provide strike locations with <12 m error for 95% of the study site

We tested the ELS methodology with a Monte Carlo simulation of CG return strokes replicated at multiple 100 m × 100 m grid points superimposed on a map of BCI and surrounding areas (Figure 9). The arrival time of the radiation from each stroke at two sensors was estimated with normally distributed error, and the resulting hyperbolic curves provided a distribution of possible return stroke locations. The actual return stroke location was determined from a recorded image of the attachment point of the stroke to the forest canopy. The horizontal pixel value of the strike point in the image provided location data (again, with normally distributed error) relative to the field of view and azimuth of the camera lens, thus generating the requisite third point of reference for triangulation of the strike location. Comparison of the estimated return stroke locations with their actual locations from the simulation showed that, once installed, the ELS system will yield <12 m error in strike locations over 95% of the study site (Figure 9).

### 2.3 Field assessment

We located, identified, and assessed the condition of struck trees and lianas in each strike location following established guidelines (USDA 1999). We recorded up to 25 different pieces of information about each strike location and sketched the spatial distribution of damage. When necessary, we used the single‐rope technique to climb nearby undamaged trees (Perry, 1978) to verify ground‐based observations. Trees not on established forest research plots were measured (as diameter at breast height; dbh) and identified from leaf samples. Each strike location was surveyed as soon as possible after the event, and again 3–4 months later to document mortality and the production of coarse woody debris (CWD). We quantified CWD volume at each strike location by tallying all dead trees >10 cm dbh, crown dieback, and dead liana stems >2 cm dbh. The amount of CWD attributable to crown dieback was estimated based on general tree architecture and associated patterns of allometry in crown area (Bohlman & O'Brien, 2006; Chave et al., 2014; Malhi, Baldocchi, & Jarvis, 1999; Montgomery & Chazdon, 2001; O'Brien, Hubbell, Spiro, Condit, & Foster, 1995). Although rough, these approximations provided an overall estimate of CWD production for each strike location, which will provide a basis for comparison with ongoing CWD inventories in the forest dynamics plots on BCI.

To generate a list of diagnostic cues for the post hoc identification of lightning damage, we qualitatively compared patterns of vegetation damage in strike sites with haphazardly selected control trees (>10 cm dbh) located in patches of forest with no known recent lightning strikes. In total, we surveyed 5,000 control canopy and subcanopy trees distributed across BCI and Gigante. These surveys focused on flashover damage as the primary diagnostic cue (described below), because it was the only cue observable at every known strike site.

## 3 RESULTS

### 3.1 Recorded lightning flashes

As of October 2016, the camera‐based monitoring system recorded 91 lightning strikes to the BCI forest and surrounding mainland (e.g., Figures 5 and 6). The majority of these CG flashes (80%) were captured by only one camera in the network, and the precise strike location could not be determined in these cases. Due in part to atypically dry wet seasons of 2014 and 2015 driven by a strong ENSO cycle (Sánchez‐Murillo, Durán‐Quesada, Birkel, Esquivel‐Hernández, & Boll, 2017), we recorded and located only four CG flashes produced by just three storms that passed over BCI when at least two cameras were fully functional (i.e., we recorded at least one flash per storm within just 15% of the area of BCI). By contrast, during the first 4 months of the 2016 wet season, the cameras captured 72 lightning strikes to BCI trees from 25 storms. Nine of these flashes were recorded by more than one camera, and all nine strike locations were located and surveyed within days of the event.

Because data collection is ongoing, here we provide three examples of recorded lightning strikes that represent the range of effects observed to date. The first two example strikes occurred within 1 s of each other on 14 August 2014 (Figure 5; Video S1, Supporting Information). The third example was triangulated from three tower cameras on 21 September 2015 (Figures 6 and 7).

**Figure 10:**
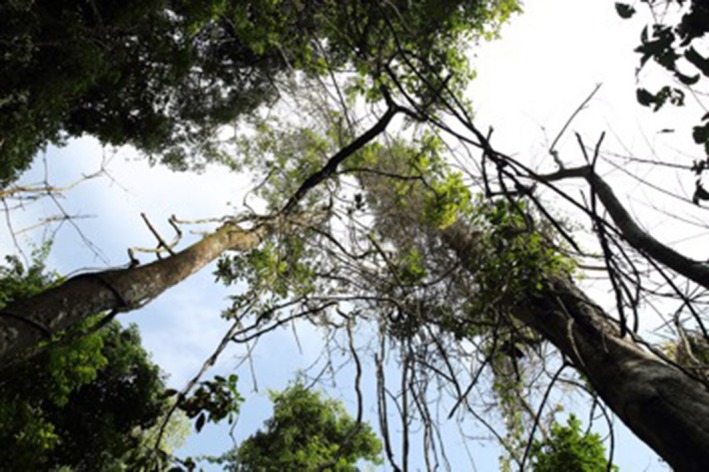
Ground‐based view of vegetation damage from the strike described as *Example 1* in the text. This image was taken 12 months poststrike. The focal tree is the large standing dead trunk ascending from the lower right corner. Note the dangling dead liana stems (lower half of the image) and the dead sapling crowns on the perimeter of the image. The green foliage in the center of the image is a portion of the liana that survived the strike

*Example 1*—A mature *Beilschmiedia pendula* tree was killed by the flash shown in the two left panels of Figure 5 (see Figure 10). A large unidentified liana inhabiting the crown of this tree was severely damaged by the discharge, but was still alive 2.5 years later. This flash killed four other trees and conspicuously damaged 10 other trees at the site.

**Figure 11:**
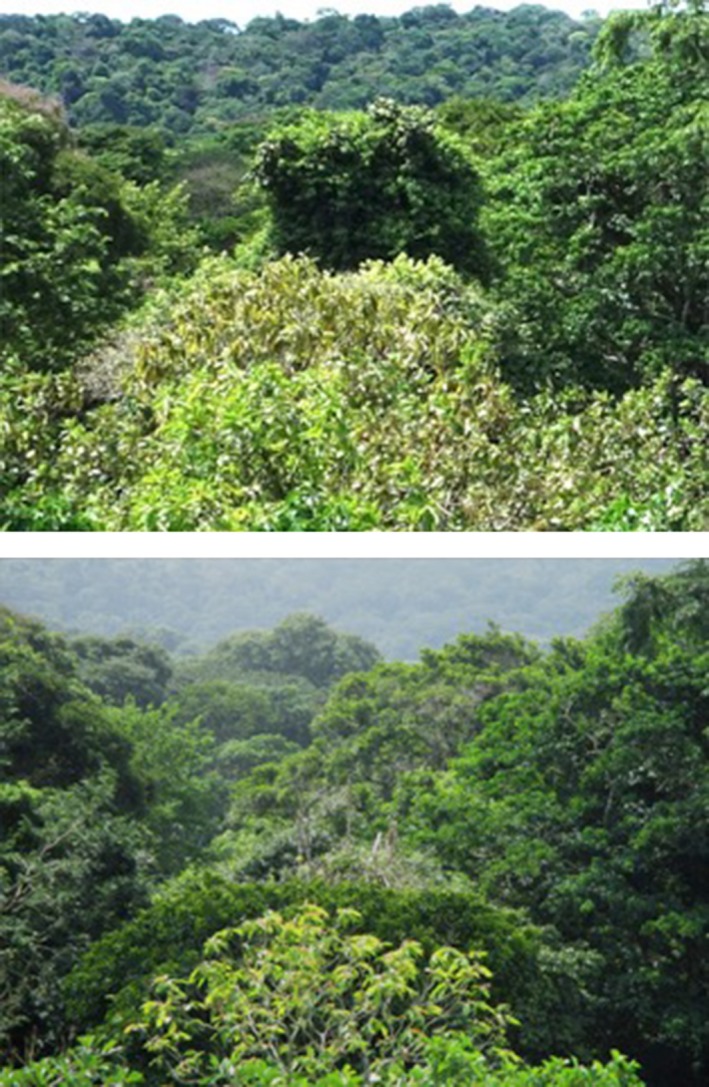
A *Sterculia apetala* tree crown with an *Arrabidaea* sp. liana 14 months before (top image, June 2014) and 3 days after (bottom image, August 2015) the lightning strike described as *Example 2* in the text. The tree crown and the liana were badly damaged, but were still living 2.5 years poststrike. No evidence of this strike or the significant crown damage was visible from the ground

*Example 2*—A mature *Sterculia apetala* tree was damaged by the larger fork of the flash shown in the two right panels of Figure 5. Multiple large branches in the upper crown of the tree were shattered, and a large *Arrabidaea* sp. liana in the crown was severely damaged (Figure 11). However, there was no evidence of the strike on the trunk of the tree or on the ground in the immediate vicinity of the tree. Thus, this event would be completely unnoticed by a ground‐based observer. Now, ca. 2.5 years later, the focal tree and the liana are alive and have extensive regrowth. No trees were killed by this event, and only one other tree was damaged.

**Figure 12:**
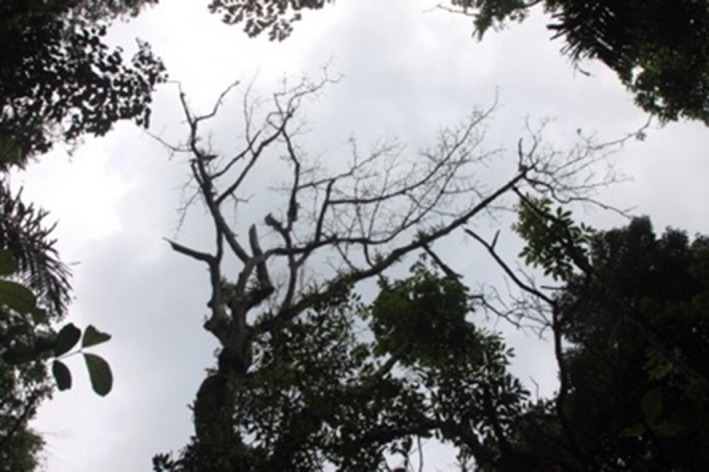
Ground‐based image of the focal tree (*Ficus obtusifolia)* in the lightning strike described as *Example 3* in the text. This photograph was taken 8 months after the strike. Note that many vascular epiphytes and epiphytic ferns are alive in the tree crown. By 12 months poststrike, all branches had fallen such that only the main trunk remained standing

*Example 3*—A large *Ficus obtusifolia* (dbh = 154 cm) and 18 other trees were damaged by this lightning flash (Figures 6 and 12). One large canopy tree died within 2 weeks of the flash and another 6 trees (>10 cm) died over the following 10 months. In addition to the direct effects of lightning, many thousands of beetles attacked three of the lightning‐damaged trees, likely contributing to their deaths. Although the damage caused by this event was extensive, the lightning flash did not conspicuously fracture branches or cause observable trunk damage.

### 3.2 Field assessment

Based on surveys of 20 strike sites, we generated a list of standardized diagnostic cues that can be used to identify lightning damage post hoc in tropical forests (Table 2). We also used observations from multiple visits to each site to approximate the time period when each lightning diagnostic cue was typically observable (Table 2). Finally, we measured the frequency of occurrence for each diagnostic cue (Table 2) using data only from strikes that were captured on two or more cameras.

**Table 2:** Diagnostic characteristics of lightning‐caused tree damage in central Panama. *N* = number of known strike sites recorded on two or more cameras adjusted based on the presence of requisite organisms for each characteristic; Frequency = fraction of known strike sites exhibiting a given characteristic; First Observed = range of time postflash that each characteristic first becomes clearly evident; Persistence = approximate duration postflash that each characteristic remains observable

Characteristic	*N*	Frequency (%)	First observed	Persistence
Flashover damage among foliage	11	100	1–4 weeks	Years
Lianas damaged	11	91	1–4 weeks	Years
Hemiepiphytes scorched and wilting	6	67	1–4 weeks	3–6 months
Beetle infestation	11	55	2–4 months	Years
Epiphytes scorched	5	0	1–4 weeks	2–4 months
Lightning scars on trunk or branches	11	0	0 days	Years

**Figure 13:**
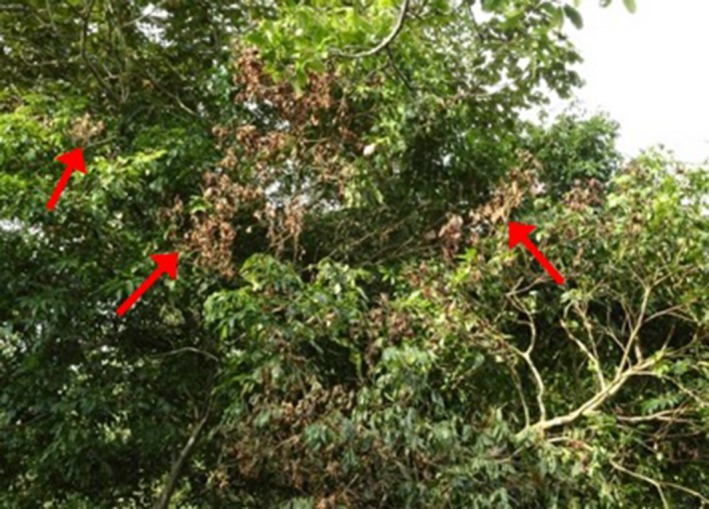
Flashover damage appears as scattered patches of dead and wilting foliage (arrows) in nearby branches of neighboring trees

**Figure 14:**
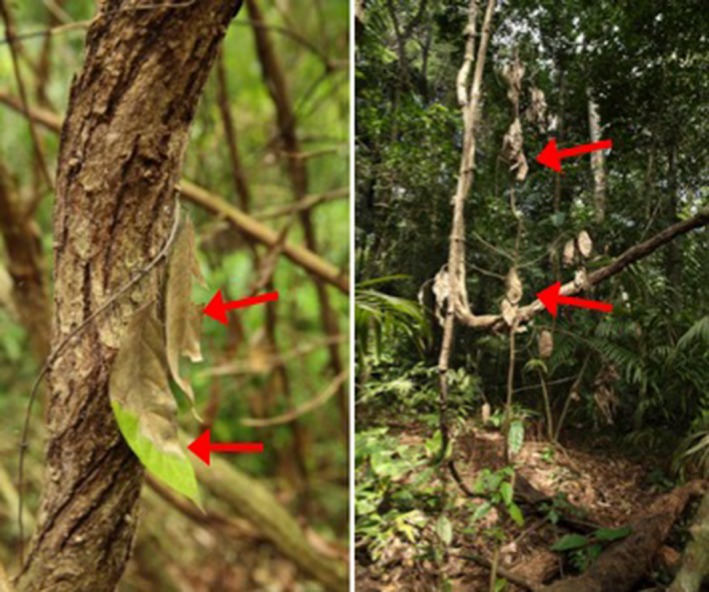
Lightning damage often appears as dead and wilting foliage (arrows) on understory plants growing in close proximity to mature lianas that have grown into or near the crown of the struck tree

The most consistent post hoc diagnostic cue of a lightning strike event was flashover damage (i.e., directionally biased vegetation mortality caused when electric current jumped from one branch to another across an air gap; Table 2). Whereas tree trunks exhibited no conspicuous damage such as lightning scars (Taylor, 1965), flashover damage occurred in all strike sites. Flashover damage was easily recognized as clusters of dead vegetation on opposite sides of gaps between neighboring plants (Figure 13), distributed radially but asymmetrically from the central struck tree, and decreasing in magnitude with increasing distance. Dead leaves and branches occurred only among individual plants that were in close proximity to each other (<1 m), such that large portions of the understory were unaffected in areas between damaged canopy trees. The distribution of flashover damage was more complicated when lianas were present, as they tended to conduct current and damage plants tens of meters from the struck tree (Figure 14). Flashover damage occurred on all types of vegetation, including adult trees, saplings, lianas, hemiepiphytes, epiphytes, and palms. The central portions of trees having flashover damage often survived for months or longer, but palms consistently died within weeks of the strike when located up to 2 m from the nearest damaged tree or liana stem.

**Figure 15:**
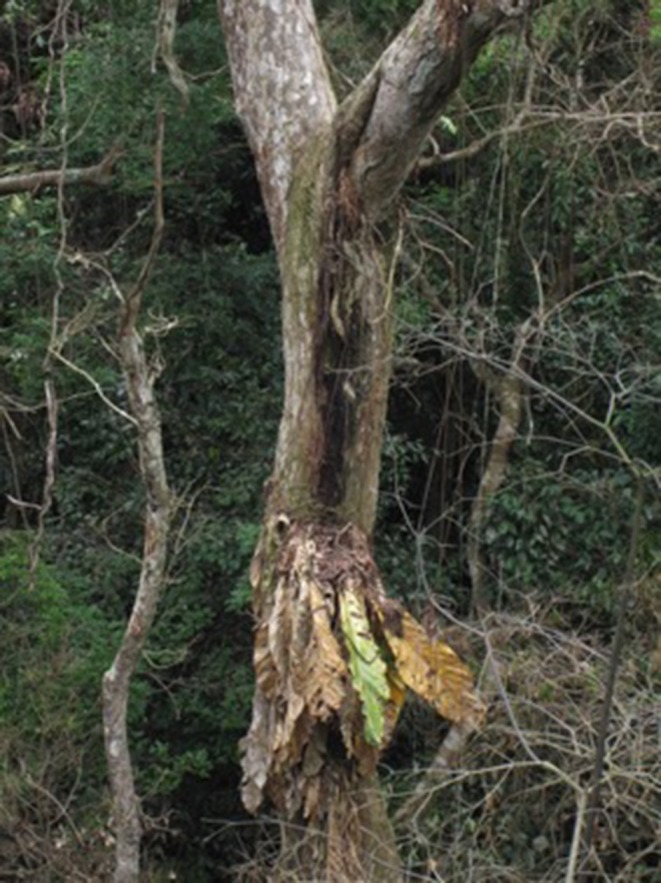
Typical lightning damage to a hemiepiphytic *Anthurium* sp. in Panama This photograph was taken ca. 4 weeks after the strike, which killed the focal *Tabebuia guayacan* and multiple nearby saplings in May 2012

In conjunction with flashover damage, damaged lianas, wilting hemiepiphytes, and beetle infestations were diagnostic of lightning strike sites (Table 2). Lianas exhibited crown dieback in patterns similar to those described for trees above. Hemiepiphytes generally wilted and died within the first 6 weeks poststrike (Figure 15). Colonizing beetles and their associated damage (accumulations of trunk entry holes and sawdust) were common ca. 2‐4 months poststrike. Vascular epiphytes generally were unharmed by lightning, but epiphytic ferns occasionally exhibited blackening of their leaves during the first 3 months poststrike, particularly when they were positioned between the host tree and a neighboring stem (i.e., in a flashover pathway). In all cases, the indiscriminate distribution of damage among plant taxa and growth forms, in combination with clear flashover patterns, served as reliable indicators of a lightning strike despite the absence of conspicuous trunk damage on any trees at a site.

Comparison with dead and damaged vegetation in control surveys showed that the diagnostic characteristics described above are reliable for ground‐based lightning damage identification. Specifically, of the 5,000 haphazardly selected canopy and subcanopy trees surveyed, damage resembling flashover among neighboring trees and other plants was exceedingly rare; we never observed evidence resembling flashover damage among more than three individual plants. We therefore suggest that post hoc field identification of lightning in the absence of camera images requires at least four individual plants that exhibit flashover damage, preferably in combination with another diagnostic cue described above.

## 4 DISCUSSION

Here, we describe a hybrid electronic sensor/camera‐based system for locating lightning‐caused disturbance in near real time, and we provide a recipe for the reliable post hoc identification of lightning damage to tropical trees, lianas, and other forest components. In concert with each other, these two methods enable the accurate quantification of lightning‐caused tree damage and mortality in forests at large spatial scales. Such data will provide an unprecedented opportunity to assess the role of lightning as an ecological disturbance at multiple levels of biological organization (Table 3). The monitoring system described here could be established in any forest with a canopy that can be viewed from at least two emergent points (e.g., towers, buildings, or exposed ridges). Given recent growth in abundance of communication towers in forests worldwide, this is fast becoming a relatively minor logistical hurdle.

**Table 3:** Example research questions that could be answered at three different levels of organization given sufficient lightning strike location data at the landscape scale

*Individual & Population*
1. Are some tree species more attractive to lightning than others?
2. Do some trees have traits that resist the damaging effects of lightning (Gora et al. unpub.)?
3. How do nonlethal lightning strikes affect tree fitness correlates (e.g., lifespan, total reproductive output, herbivore damage)?
4. Is there a relationship between tree condition at the time of a strike and the amount of damage that occurs?
5. Does lightning‐caused mortality mediate competition among trees?
*Community*
1. What is the role of lightning disturbance in gap‐phase forest dynamics (Brokaw, 1985)?
2. Do lightning strikes modify the structure of local soil microbial communities?
3. What are the effects of lightning strikes on nontree forest components (e.g., lianas, epiphytes, endophytic fungi)?
4. Do lianas protect trees against lightning damage (Yanoviak, 2013; Gora et al. unpub.)?
5. What are the effects of lightning strikes on tree inhabitants, especially ants?
6. Are lightning‐damaged trees specifically attractive to certain types of fungi or herbivores?
*Ecosystem*
1. How much carbon (as coarse woody debris) is produced by lightning per hectare?
2. How does the ecological importance of lightning change with ecosystem type?
3. Do lightning gaps differ from other types of disturbance in terms of local environmental conditions?
4. Does the amount of lightning damage vary predictably with landscape‐scale changes in soil moisture or composition?
5. How do tall metal towers and other structures affect the landscape‐scale distribution of lightning damage?

Preliminary data from this system indicate that lightning is an important agent of tropical tree mortality; indeed, lightning damages and kills more trees in the BCI forest than we anticipated at the start of the project. Most importantly, that mortality is not obviously due to lightning and is unlikely to be attributed to lightning during normal forest surveys without prior knowledge of the event. As a case in point, *Example 3* above was included in the 2015 survey of the 50 ha forest dynamics plot on BCI (Hubbell & Foster, 1983), but only the focal tree was dead at the time of the survey and the agent of mortality was not identified. Given rapid changes at this site over the 12 months poststrike, a 2016 plot survey likely would have recorded the agent of mortality for all trees killed at this site as blowdown. Thus, it is clear that lightning damage is easily overlooked or misidentified in practice.

Once the monitoring system described here has been established in a forest, the number of large‐scale, integrative ecological questions that can be answered immediately increases (Table 3). In particular, measuring the ecosystem‐level relevance of lightning‐caused disturbance requires accurate information about its effects on individual trees and tree species on a forest‐wide scale, and this system is the first to provide such information with a high degree of accuracy. Specifically, this network overcomes two general and very pertinent problems overshadowing all historical research concerning the ecological effects of lightning: (1) overwhelming dependence on anecdotal and post hoc observations and (2) a general lack of information concerning lightning characteristics (intensity, polarity, and flash duration). Post hoc assessments of lightning damage in forests are biased toward detection of conspicuous evidence. As described for *Example 3* above, such evidence is easily overlooked, at least in tropical forests.

Given the estimated flash frequency for BCI described above (i.e., 150 CG flashes per year), the number of strikes recorded by at least one camera in the network since 2014 is much lower than expected. Specifically, all iterations of the network provided ca. 50% coverage of BCI by at least one camera, suggesting that >200 strikes should have been recorded since 2014. The reasons for this difference are twofold. First, due to logistical constraints (including humidity and lightning damage to equipment), the cameras were not operating continuously during all months of each wet season. Cumulative recording time was ca. 2 months in 2014, 4 months in 2015, and 5 months in 2016. Second, due to a strong ENSO event (Sánchez‐Murillo et al., 2017), the field site did not experience any electrically active storms during 4 of the 6 months that the system was active during the 2014 and 2015 wet seasons. Finally, although the variety of strikes we recorded indicates that most flashes are captured by this system, it is impossible to test for false negatives using only video cameras. We expect that factors limiting the consistency and effectiveness of this system will be overcome by the deployment of the ELS system and advances in remote sensing and video technologies.

Finally, growth in telecommunications infrastructure has led to an abundance of towers in forests. Such towers may function as lightning attractors; they are often on hilltops, extend well above the forest canopy, and are equipped with lightning rods. Rakov and Uman (2003, p. 51) estimated that a tower with an effective height of 60 m (i.e., exceeding the heights of surrounding objects by 60 m) in an area with 10 CG flashes km^−2^ year^−1^ will get struck once every two years. Thus, if lightning plays a significant role in forest dynamics, such towers are potentially changing those dynamics. Coincidentally, canopy cranes used around the world to study forest ecology (e.g., Parker, Smith, & Hogan, 1992) likely have similar effects. The study site for this project has both high lightning frequency and a relatively high concentration of metal towers extending >10 m above the surrounding canopy. Our observations are too preliminary to evaluate the effects of these towers on the frequency of lightning damage to nearby trees, but the monitoring system described in this study is generating data that eventually will resolve this issue.

## 5 CONCLUSIONS

Here, we demonstrate a relatively simple and inexpensive system that provides accurate lightning strike location and flash characteristic information in real time (within minutes to hours) and over large spatial scales. Consequently, this system provides an unprecedented opportunity to track the effects of both conspicuous and inconspicuous lightning damage, thus enabling structured and unbiased tests of hypotheses related to the ecological effects of lightning for the first time. Moreover, by pairing this system with a well‐studied forest, such as a forest dynamics plot, it is possible to conduct long‐term and detailed investigations using historic information about monitored individual trees. Because the ecological effects of lightning are largely unstudied, we expect that increased use of this and similar systems will lead to the initiation of many unanticipated avenues for future research.

## CONFLICT OF INTEREST

None declared.

## AUTHOR CONTRIBUTIONS

All authors assisted with the installation, implementation, and maintenance of the camera and/or ELS networks. SPY collected field data, wrote the manuscript, and managed the project. EMG collected field data, processed videos, and managed field operations. JMB assisted in the development of the camera and ELS networks, processed videos, and composed relevant computer code. PMB conceived, developed, and tested the ELS network; composed relevant computer code; and managed the project. MD collected field data, processed videos, and composed relevant computer code. All authors contributed critically to the drafts and gave final approval for publication.

## References

[ece33095-bib-0001] Albrecht, R. I. , Goodman, S. J. , Buechler, D. E. , Blakeslee, R. J. , & Christian, H. J. (2016). Where are the lightning hotspots on Earth? Bulletin of the American Meteorological Society, 97, 2051–2068.

[ece33095-bib-0002] Anderson, J. A. R. (1964). Observations on climatic damage in peat swamp forest in Sarawak. Commonwealth Forestry Review, 43, 145–158.

[ece33095-bib-0003] Andrews, C. J. , Cooper, M. A. , Darveniza, M. , & Mackerras, D. (1992). Lightning injuries: Electrical, medical, and legal aspects (p. 208). Boca Raton, FL: CRC Press.

[ece33095-bib-0004] Anonymous, (1898). Which trees attract lightning? Monthly Weather Review, 26, 257–258.

[ece33095-bib-0005] Bitzer, P. M. (2017). Global distribution and properties of continuing current in lightning. Journal of Geophysical Research: Atmospheres, 122, 1033–1041.

[ece33095-bib-0006] Bitzer, P. M. , Christian, H. J. , Stewart, M. , Burchfield, J. , Podgorny, S. , Corredor, D. , … Franklin, V. (2013). Characterization and applications of VLF/LF source locations from lightning using the Huntsville Alabama Marx Meter Array. Journal of Geophysical Research: Atmospheres, 118, 3120–3138.

[ece33095-bib-0007] Boccippio, D. J. , Cummins, K. L. , Christian, H. J. , & Goodman, S. J. (2001). Combined satellite‐and surface‐based estimation of the intracloud‐cloud‐to‐ground lightning ratio over the continental United States. Monthly Weather Review, 129, 108–122.

[ece33095-bib-0008] Bohlman, S. , & O'Brien, S. (2006). Allometry, adult stature and regeneration requirement of 65 tree species on Barro Colorado Island, Panama. Journal of Tropical Ecology, 22, 123–136.

[ece33095-bib-0009] Botley, C. M. (1951). The lightning discharge in myth and legend. Weather, 6, 277–279.

[ece33095-bib-0010] Bouquegneau, C. , & Rakov, V. (2010). How dangerous is lightning?. Mineola, NY: Dover Publications.

[ece33095-bib-0011] Brokaw, N. V. L. (1985). Gap‐phase regeneration in a tropical forest. Ecology, 66, 682–687.

[ece33095-bib-0012] Brünig, E. F. (1964). A study of damage attributed to lightning in two areas of *Shorea albida* forest in Sarawak. Commonwealth Forestry Review, 43, 134–144.

[ece33095-bib-0013] Chave, J. , Réjou‐Méchain, M. , Búrquez, A. , Chidumayo, E. , Colgan, M. S. , Delitti, W. B. C. , … Vieilledent, G. (2014). Improved allometric models to estimate the aboveground biomass of tropical trees. Global Change Biology, 20, 3177–3190. 24817483 10.1111/gcb.12629

[ece33095-bib-0014] Christian, H. J. , Blakeslee, R. J. , Boccippio, D. J. , Boeck, W. L. , Buechler, D. E. , Driscoll, K. T. , … Stewart, M. F. (2003). Global frequency and distribution of lightning as observed from space by the Optical Transient Detector. Journal of Geophysical Research, 108, 4005.

[ece33095-bib-0015] Cochrane, M. A. (2003). Fire science for rainforests. Nature, 421, 913–919. 12606992 10.1038/nature01437

[ece33095-bib-0016] Coulson, R. N. , Hennier, P. B. , Flamm, R. O. , Rykiel, E. J. , Hu, L. C. , & Payne, T. L. (1983). The role of lightning in the epidemiology of the Southern Pine Beetle. Zeitschrift für angewandte Entomologie, 96, 182–193.

[ece33095-bib-0017] Croat, T. B. (1978). Flora of Barro Colorado Island. Stanford, CA: Stanford University Press.

[ece33095-bib-0018] Cummins, K. L. , & Murphy, M. J. (2009). An overview of lightning locating systems: History, techniques, and data uses, with an in‐depth look at the U.S. NLDN. IEEE Transactions on Electromagnetic Compatibility, 51, 499–518.

[ece33095-bib-0019] DuCharme, E. P. (1972). Lightning and decline of citrus trees in Florida groves. Proceedings of the Florida State Horticultural Society, 85, 80–84.

[ece33095-bib-0020] Fernando, M. , Mäkelä, J. , & Cooray, V. (2010). Lightning and trees In Cooray V. (Ed.), Lightning protection (pp. 843–858). London: Institution of Engineering and Technology.

[ece33095-bib-0021] Franklin, B. (1769). Experiments and observations on electricity, made at Philadelphia in America. London: David Henry.

[ece33095-bib-0022] Fuquay, D. M. , Taylor, A. R. , Hawe, R. G. , & Schmid, C. W. Jr (1972). Lightning discharges that caused forest fires. Journal of Geophysical Research, 77, 2156–2158.

[ece33095-bib-0023] Furtado, C. X. (1935). Lightning injuries to trees. Journal of the Malaysian Branch of the Royal Asiatic Society, 13, 157–162.

[ece33095-bib-0024] Goldammer, J. G. , & Price, C. (1998). Potential impacts of climate change on fire regimes in the tropics based on MAGICC and a GISS GCM‐derived lightning model. Climatic Change, 39, 273–296.

[ece33095-bib-0025] Gora, E. M. , & Yanoviak, S. P. (2015). Electrical properties of temperate forest trees: A review and quantitative comparison with vines. Canadian Journal of Forest Research, 45, 236–245.

[ece33095-bib-0026] Hubbell, S. P. , & Foster, R. B. (1983). Diversity of canopy trees in a neotropical forest and implications for conservation In Sutton S. L., Whitmore T. C., & Chadwick A. C. (Eds.), Tropical rain forest: Ecology and management (pp. 25–41). Oxford: Blackwell Scientific.

[ece33095-bib-0027] Kays, R. , Tilak, S. , Crofoot, M. , Fountain, T. , Obando, D. , Ortega, A. , … Wikelski, M. (2011). Tracking animal location and activity with an automated radio telemetry system in a tropical rainforest. The Computer Journal, 12, bxr072.

[ece33095-bib-0028] Krider, E. P. (1992). On the electromagnetic fields, Poynting vector, and peak power radiated by lightning return strokes. Journal of Geophysical Research, 97(D14), 15913–15916.

[ece33095-bib-0029] Leigh, E. G. Jr , Rand, A. S. , & Windsor, D. M. (1996). The ecology of a tropical forest. Washington, DC: Smithsonian Institution.

[ece33095-bib-0030] Magnusson, W. E. , Lima, A. P. , & de Lima, O. (1996). Group lightning mortality of trees in a Neotropical forest. Journal of Tropical Ecology, 12, 899–903.

[ece33095-bib-0031] Mäkelä, J. , Karvinen, E. , Porjo, N. , Mäkelä, A. , & Tuomi, T. (2009). Attachment of natural lightning flashes to trees: Preliminary statistical characteristics. Journal of Lightning Research, 1, 9–21.

[ece33095-bib-0032] Mäkelä, A. , Mäkelä, J. , Haapalainen, J. , & Porjo, N. (2016). The verification of lightning location accuracy in Finland deduced from lightning strikes to trees. Atmospheric Research, 172–173, 1–7.

[ece33095-bib-0033] Malhi, Y. , Baldocchi, D. D. , & Jarvis, P. J. (1999). The carbon balance of tropical, temperate, and boreal forests. Plant, Cell & Environment, 22, 715–740.

[ece33095-bib-0034] Montgomery, R. A. , & Chazdon, R. L. (2001). Forest structure, canopy architecture, and light transmittance in tropical wet forests. Ecology, 82, 2707–2018.

[ece33095-bib-0035] Norris, J. R. , Allen, R. J. , Evan, A. T. , Zelinka, M. D. , O'Dell, C. W. , & Klein, S. A. (2016). Evidence for climate change in the satellite cloud record. Nature, 536, 72–75. 27398619 10.1038/nature18273

[ece33095-bib-0036] O'Brien, S. T. , Hubbell, S. P. , Spiro, P. , Condit, R. , & Foster, R. B. (1995). Diameter, height, crown, and age relationship in eight Neotropical tree species. Ecology, 76, 1926–1939.

[ece33095-bib-0037] Orville, R. E. (1968). Photograph of a close lightning flash. Science, 162, 666–667. 17736043 10.1126/science.162.3854.666

[ece33095-bib-0038] Outcalt, K. W. (2008). Lightning, fire and longleaf pine: Using natural disturbance to guide management. Forest Ecology and Management, 255, 3351–3359.

[ece33095-bib-0039] Parker, G. G. , Smith, A. P. , & Hogan, K. P. (1992). Access to the upper forest canopy with a large tower crane. BioScience, 42, 664–670.

[ece33095-bib-0040] Perry, D. R. (1978). A method of access into the crowns of emergent and canopy trees. Biotropica, 10, 155–157.

[ece33095-bib-0041] Price, C. (2009). Will a drier climate result in more lightning? Atmospheric Research, 91, 479–484.

[ece33095-bib-0042] Price, C. , & Rind, D. (1993). What determines the cloud‐to‐ground lightning fraction in thunderstorms? Geophysical Research Letters, 20, 463–466.

[ece33095-bib-0043] Price, C. , & Rind, D. (1994a). The impact of a 2 X CO_2_ climate on lightning‐caused fires. Journal of Climate, 7, 1484–1494.

[ece33095-bib-0044] Price, C. , & Rind, D. (1994b). Possible implications of global climate change on global lightning distributions and frequencies. Journal of Geophysical Research, 99, 10823–10831.

[ece33095-bib-0045] Rakov, V. A. , & Uman, M. A. (2003). Lightning: Physics and effects. Cambridge: Cambridge University Press.

[ece33095-bib-0046] Romps, D. M. , Seeley, J. T. , Vollaro, D. , & Molinari, J. (2014). Projected increase in lightning strikes in the United States due to global warming. Science, 346, 851–854. 25395536 10.1126/science.1259100

[ece33095-bib-0047] Sánchez‐Murillo, R. , Durán‐Quesada, A. M. , Birkel, C. , Esquivel‐Hernández, G. , & Boll, J. (2017). Tropical precipitation anomalies and *d*‐excess evolution during El Niño 2014‐16. Hydrological Processes, 31, 956–967.

[ece33095-bib-0048] Stall, C. A. , Cummins, K. L. , Krider, E. P. , & Cramer, J. A. (2009). Detecting multiple ground contacts in cloud‐to‐ground lightning flashes. Journal of Atmospheric and Oceanic Technology, 26, 2392–2402.

[ece33095-bib-0049] Stone, G. E. (1903). Injuries to shade trees from electricity. Hatch Experiment Station of the Massachusetts Agricultural College, 91, 1–21.

[ece33095-bib-0050] Stone, G. E. (1916). Shade trees, characteristics, adaptation, diseases and care. Bulletin of the Massachusetts Agricultural Experiment Station, 170, 123–264.

[ece33095-bib-0051] Taylor, A. R. (1964). Lightning damage to forest trees in Montana. Weatherwise, 17, 61–65.

[ece33095-bib-0052] Taylor, A. R. (1965). Diameter of lightning as indicated by tree scars. Journal of Geophysical Research, 70, 5693–5695.

[ece33095-bib-0053] Taylor, A. R. (1974). Ecological aspects of lightning in forests. Proceedings of the Tall Timbers Fire Ecology Conference, 13, 455–482.

[ece33095-bib-0054] Tutin, C. E. G. , White, L. J. T. , & Mackanga‐Missandzou, A. (1996). Lightning strike burns large forest tree in the Lope Reserve, Gabon. Global Ecology and Biogeography Letters, 5, 36–41.

[ece33095-bib-0055] USDA Forest Service (1999). Forest health monitoring 1999 field methods guide. Research Triangle Park, NC: USDA Forest Service, National Forest Health Monitoring Program.

[ece33095-bib-0056] Wadsworth, F. H. (1943). Lightning damage in ponderosa pine stands of northern Arizona. Journal of Forestry, 41, 684–685.

[ece33095-bib-0057] Williams, E. R. (1992). The Schumann resonance: A global tropical thermometer. Science, 256, 1184–1187. 17795213 10.1126/science.256.5060.1184

[ece33095-bib-0058] Williams, E. R. (2005). Lightning and climate: A review. Atmospheric Research, 76, 272–287.

[ece33095-bib-0059] Yanoviak, S. P. (2013). Shock value: Are lianas natural lightning rods? In Lowman M., Devy S., & Ganesh T. (Eds.), Treetops at risk: Challenges of global forest canopies (pp. 147–154). New York, NY: Springer.

[ece33095-bib-0060] Yanoviak, S. P. , Gora, E. M. , Fredley, J. , Bitzer, P. M. , Muzika, R.‐M. , & Carson, W. P. (2015). Direct effects of lightning in temperate forests: A review and preliminary survey in a hemlock‐hardwood forest of the northern United States. Canadian Journal of Forest Research, 45, 1258–1268.

